# Risk factors for initially missing injuries in severely injured children and adolescents: a retrospective study from the traumaregister DGU

**DOI:** 10.1038/s41598-025-28220-1

**Published:** 2025-11-18

**Authors:** Nikos Karvouniaris, Timo Riesle, Ferdinand C. Wagner, Rolf Lefering, Hagen Schmal, Jörg Bayer

**Affiliations:** 1 Department of Orthopaedic and Trauma Surgery, Faculty of Medicine, Medical Center, University of Freiburg, Hugstetter Straße 55, 79106 Freiburg, Germany; 2 Department of Orthopaedic and Trauma Surgery, Schwarzwald-Baar Hospital, Klinikstraße 11, 78052 Villingen-Schwenningen, Germany; 3 Faculty of Health, IFOM-Institute for Research in Operative Medicine, University Witten/Herdecke, Ostmerheimer Straße 200, 51109 Cologne, Germany; 4 Department of Orthopedic Surgery, University Hospital Odense, Sdr. Boulevard 29, 5000 Odense, Denmark

**Keywords:** Risk factors, Paediatrics, Surgery

## Abstract

Injuries diagnosed after the emergency department period are a significant challenge to trauma care, since these delayed diagnosed injuries (DDI) may require specific additional treatment. The aim of this study was to obtain an overview of DDI in children and adolescents and to meticulously analyze the underlying reasons leading to initially missing injuries by evaluating data from the TraumaRegister DGU. TraumaRegister DGU data from the years 2010 to 2021 were evaluated. All patients up to the age of 20 were included. Patients older than 20 years of age and patients who died in the trauma room were excluded. All injuries diagnosed after the trauma room period were included in the statistics and defined as DDI, since we focus on the initial care phase and stress that injuries can be overlooked initially, but should be discovered during in hospital treatment. A total of 12,733 patients were included in this study, of whom 68.5% were male. In 1,246 patients (9.8%), at least one diagnosis was a DDI. Patients with DDI had an average age of 15.1 ± 5.3 years and had a longer stay in the intensive care unit with 9.4 ± 11.6 days than those without an initially missed injury (no-DDI) with 5.6 ± 9.1 days. The DDI-group’s mean ISS was 24.0 ± 14.5 and thus substantially higher than the mean ISS in the no-DDI group (17.5 ± 12.2). Independent risk factors for DDI were number of diagnoses per patient (OR 1.19; CI 1.16–1.22, *p* < 0.001), hospital level 2/3 (OR 1.89; CI 1.61–2.22, *p* < 0.001), relevant (AIS ≥ 2) injuries to the abdomen (OR 1.23; CI 1.06–1.42, *p* = 0.006) or lower extremities (OR 1.25; CI 1.08–1.43, *p* = 0.002). The present study demonstrates that injuries in pediatric and adolescent trauma patients are frequently missed initially during the context of trauma room treatment and diagnostics. These DDIs have an impact on the length of hospital and intensive care unit stay. We identified risk factors for DDI, i.e. higher numbers of trauma diagnoses, a higher injury severity score and treatment in level-2 or -3 trauma centers.

## Introduction

Management of pediatric trauma remains a challenge in both pre-hospital settings and emergency departments. While minor injuries among pediatric patients are frequent, life-threatening injuries in children and adolescents are rare and pediatric polytrauma patients can exhibit diverse injury patterns and clinical characteristics^1,2^. Seriously injured children are particularly common in road traffic accidents. According to the German Federal Statistical Office, in 2018 an average of one child under the age of 15 was involved in a road traffic accident every 18 min^4^. Adequate management of severely and critically injured children requires knowledge of common injury patterns, incidence, mortality, and differences between pediatric and adult injuries^5^. The assessment protocols for pediatric trauma care implemented in the hospital trauma room follow the standard of Advanced Trauma Life Support (ATLS) and are designed to filter critically injured children on the basis of vital signs, and to identify and treat the most serious injuries according to the principle “treat first what kills first”^6^. However, recognizing and treating such injuries in children and adolescents at the time of initial assessment remains a major challenge. Depending on the child’s age and the severity of the injury, it may be impossible to adequately communicate pain or describe the trauma mechanism. In addition, physical examination of children can be challenging, and diagnosing and subsequent assessments of children can be more difficult, as they entail different anatomical and physiological characteristics compared to adults^7,8^. According to current guidelines for the management of polytrauma in children, once life-threatening injuries have been excluded, either a detailed follow-up ultrasound examination of the affected body regions or a CT scan should be performed, depending on the primary findings and by consensus of the trauma team, to identify all relevant injuries^9^. In polytraumatized children with relevant injuries (traumatic brain injuries (TBI) involving loss of consciousness, thoracic/abdominal or pelvic injuries, and fractures of at least 2 tubular bones), multi-slice whole-body computed tomography (WBCT) is recommended^10^. However, in severely injured children, WBCT has not been shown to have a significant effect in terms of mortality^11–13^. For adults, there is evidence that the risk for missing injuries during the initial treatment phase is increased due to certain factors such as severe TBI, the presence of life-threatening injuries or a high Injury Severity Score (ISS)^14^. A systematic classification of delayed diagnosed injuries (DDI) has been suggested that takes the time elapsed from trauma room treatment into account (groups A-C) and the therapeutic relevance (subgroups 1–3) of the injuries^15^. Whether an increasing workload and possible associated fatigue of medical staff, such as radiologists or trauma surgeons, are potentially other contributing factors to initially missing injuries has not been conclusively established yet^16^. Delayed diagnosed injuries can have significant health consequences, including long-term sequelae to the patient, and can lead to prolonged hospitalization and intensive care stay, resulting in significant additional costs to the healthcare system^17^. While the burden and risk of initially missed injuries in adult trauma patients have been repeatedly assessed^14,15^, there is a paucity of data in the multiply-injured pediatric patient population.

Therefore, the aim of this study was to evaluate initially missed injuries in children and adolescents relying on data from the TraumaRegister DGU of the German Trauma Society (DGU) and to investigate potentially associated risk factors.

## Methods

The TraumaRegister DGU (TR-DGU) of the German Trauma Society (Deutsche Gesellschaft für Unfallchirurgie, DGU) was founded in 1993 and represents a multi-center database containing pseudonymized and standardized documentation of severely injured patients. Data are collected prospectively in 4 consecutive phases: (A) pre-hospital phase, (B) trauma room and subsequent operating theatre phase, (C) intensive care unit and (D) discharge. The documentation includes detailed information on demographics, injury patterns, comorbidities, pre-hospital and clinical management, intensive care course, key laboratory findings including transfusion data, and outcome. The inclusion criterion is admission to hospital via the trauma room with subsequent monitoring in intensive or intermediate care, or arrival at hospital with vital signs and death before admission to intensive care unit.

The infrastructure for documentation, data management and data analysis are provided by the AUC - Academy for Trauma Surgery (AUC - Akademie der Unfallchirurgie GmbH), which is affiliated to the German Trauma Society. The scientific leadership is provided by the Committee on Emergency Medicine, Intensive Care and Trauma Management (Sektion NIS) of the German Trauma Society. Participating clinics enter their pseudonymized data into a central database via a web-based application. Scientific analyses are approved according to a peer review process defined in the publication guidelines of the TR-DGU. The participating clinics are mainly located in Germany (90%), but an increasing number of clinics from other countries are also contributing data (currently from Austria, Belgium, China, Finland, Luxembourg, Slovenia, Switzerland, the Netherlands and the United Arab Emirates). Currently, about 28,000 cases from almost 700 clinics are added to the database each year. Participation in the TR-DGU is on a voluntary basis. It is mandatory for TraumaNetzwerk DGU^®^ clinics to enter at least one basic data set for quality assurance purposes.

This retrospective multicenter cross-sectional study evaluated the data on all severely injured children and adolescents up to 20 years old in the TR-DGU from 01.01.2010 to 31.12.2021. The study strictly adheres to the publication guidelines of the TR-DGU and is registered under the TR-DGU project ID 2023-001. The research project has been approved by our local ethics committee (University of Freiburg Ethics Committee, 23-1372-S1-retro). Due to the retrospective nature of the study, University of Freiburg Ethics Committee waived the need of obtaining informed consent. All experiments were performed in accordance with relevant guidelines and regulations.

For this study we retrospectively analyzed the existing TR-DGU dataset epidemiologically and statistically to extract relevant data. The data was then filtered based on strict inclusion and exclusion criteria, with age being the determining factor. Inclusion criteria were set at age ≤ 20 years. Only data from hospitals in Germany were included. The basic data entered was used for analysis, with no additional requirements from quality management (QM) systems. Patients transferred early (within 48 h after admission) to another facility and all patients who died in the trauma room were excluded from further analysis. Emergency trauma care in certified German trauma centers in general follows the ATLS algorithm and staff consists of at least a board-certified surgeon with expertise in pediatric trauma care and a board-certified anesthesiologist, according to published guidelines^9^.

Delayed diagnosed injuries (DDI) were the primary target and defined as all injuries being diagnosed after the initial trauma room phase during subsequent examinations in the intensive care unit or regular ward. Secondary outcome measures included mortality, ventilation days and length of intensive care unit/hospital stay.

Since our investigation relies on retrospective patient data documented by the time of hospital discharge, the term “missed injury” reports an injury detected after trauma room discharge, but still during the ensuing in-hospital treatment.

The terms missed injuries and delayed diagnosed injuries are often used interchangeably in the literature, as both refer to diagnostic delays in recognizing injuries. While the term “missed” refers to an injury that was completely overlooked during the examination and treatment, “delayed diagnosed” injury describes a delayed diagnosis made later, even despite initial indications, i.e., for a radiological examination, or that developed secondarily due to worsening of the initial injury. Additionally, the term delayed diagnosed injury also accounts for diagnoses that could not be specified during initial workup due to missing indication to intensify diagnostics during early patient treatment (e.g. magnetic resonance imaging).

In this study, we chose the term delayed diagnosed injury as it focuses on the initial care phase and stresses that injuries can be overlooked in the initial care of seriously injured children due to time pressure, the injuries’ complexity, or the prioritization of life-threatening conditions, but they should be discovered during in hospital treatment.

### Statistical analysis

Statistical analysis was conducted using SPSS (Version 29, IBM Inc., Armonk, NY, USA). Continuous variables are presented as mean ± standard deviation (SD), while prevalence rates are presented as percentages. To identify potential risk factors for the presence of DDI a multivariable logistic regression analysis was performed. Due to missing datasets, 17 patients had to be excluded from the regression analysis. Potential predictors (continuous: total number of diagnoses; categorical: transfer-in, hospital level of care, high injury severity, injured body region, unconsciousness (GCS ≤ 8), computed tomography performed, age group) were analyzed for the dependent event “at least one delayed diagnosed injury/initially missed injury”. For categorical variables results are reported as odds ratios (OR) relative to a reference category. A category with an OR > 1.00 makes the presence of a delayed injury more probable than in patients with the reference category. OR were reported with a 95% confidence interval (CI95).

A p-value < 0.05 was considered statistically significant.

## Results

The TR-DGU database of the years 2010–2021 encompasses overall 20,116 children aged 0–15 years and 32,183 adolescents aged 16–20 years. After application of inclusion and exclusion criteria we state our results based on a population of 4,814 children aged 0–15 years and 7,919 adolescents aged 16–20 years, totaling 12,733 patients. The majority were male patients (68.5%). A total of 60,100 diagnoses were made, including 1809 (3%) delayed diagnoses. On the patient level, 9.8% of the patients (*n* = 1246) had at least one initially missed injury. Comprehensive demographic data is shown in Table 1.

**Table 1:** Demographic data.

	DDI group *N* = 1246	No-DDI group *N* = 11,487	Overall *N* = 12,733
General Data			
Age groups (in years)			
Overall (mean ± SD)	15.1 ± 5.3	14.6 ± 5.5	14.6 ± 5.5
0–5	121 (8.9%)	1231 (91.1%)	1352
6–10	112 (8.4%)	1219 (91.6%)	1331
11–15	168 (7.9%)	1963 (92.1%)	2131
16–20	845 (10.7%)	7074 (89.3%)	7919
Number of diagnoses	6.6 ± 3.8	4.5 ± 2.9	4.7 ± 3.0
Length of hospital stay (days)	18.8 ± 17.5	13.9 ± 15.5	14.4 ± 15.7
Length of ICU stay (days)	9.4 ± 11.7	5.6 ± 9.1	6.0 ± 9.5
Hospital Level of Trauma Care: 1 2 3	1008 (80.9%) 187 (15.0%) 51 (4.1%)	9908 (86.3%) 1279 (11.1%) 300 (2.6%)	10,916 (85.7%) 1466 (11.5%) 351 (2.8%)
Transferred patients	113 (9.1%)	1197 (10.4%)	1310 (10.3%)
Night-time admission	633 (50.8%)	5491 (47.8%)	6124 (48.1%)
Weekend admission	586 (47.0%)	5556 (48.4%)	6142 (48.1%)
Clinical Data			
Prehospital intubation	477 (38.3%)	3023 (26.3%)	3500 (27.5%)
PRBC administration in the trauma room	167 (13.4%)	943 (8.2%)	1246 (9.8%)
BP ≤ 90mmHg pre-hospital	161 (12.9%)	1041 (9.1%)	1202 (9.4%)
BP ≥ 90mmHg pre-hospital	131 (10.5%)	904 (7.9%)	1035 (8.1%)
Injury severity (ISS)	24.1 ± 14.5	17.5 ± 12.2	18.2 ± 12.6
Emergency surgery	381 (30.6%)	2988 (26.0%)	3369 (26.5%)
Whole-body computed tomography scan (WBCT)	948 (76.1%)	8308 (72.3%)	9256 (72.7%)
Unconsciousness (GCS ≤ 8)	322 (25.8%)	1837 (16.0%)	2159 (17.0%)
Polytrauma	341 (27.4%)	1655 (14.4%)	1996 (15.7%)
ISS 1–8	119 (9.6%)	2391 (20.8%)	2510 (19.7%)
ISS 9–15	264 (21.2%)	3450 (30.0%)	3714 (29.2%)
ISS 16–24	325 (26.1%)	2913 (25.4%)	3238 (25.4%)
ISS 25–34	303 (24.3%)	1782 (15.5%)	2085 (16.4%)
ISS 35–49	153 (12.3%)	620 (5.4%)	773 (6.1%)
ISS 50–74	63 (5.1%)	271 (2.4%)	334 (2.6%)
ISS 75	19 (1.5%)	60 (0.5%)	79 (0.6%)
Deceased in hospital	74 (5.9%)	509 (4.4%)	583 (4.5%)
Mechanism of injury			
Car	440 (35.3%)	3054 (26.6%)	3494 (27.4%)
Motorcycle	244 (19.6%)	1959 (17.0%)	2203 (17.3%)
Bicycle	94 (7.5%)	1153 (10.0%)	1247 (9.8%)
Walker	116 (9.3%)	1253 (10.9%)	1369 (10.8%)
Highfall	154 (12.4%)	1597 (13.9%)	1751 (13.8%)
Lowfall	76 (6.1%)	978 (8.5%)	1054 (8.2%)
Other	102 (8.2%)	1342 (11.7%)	1444 (11.3%)
Blunt trauma	1173 (94.1%)	10,676 (92.9%)	11,849 (93.1%)
Injury severity by body region			
AIS Abdomen 2+	409 (32.8%)	2489 (21.7%)	2898 (22.8%)
AIS Spine 2+	343 (27.5%)	2671 (23.3%)	3014 (23.7%)
AIS Arms 2+	469 (37.6%)	3267 (28.4%)	3736 (29.3%)
AIS Legs 2+	493 (39.6%)	3521 (30.7%)	4014 (31.5%)
AIS Pelvis 2+	257 (20.6%)	1895 (16.5%)	2152 (16.9%)
AIS Head 2+	733 (58.8%)	5591 (48.7%)	6324 (49.7%)
AIS Thorax 2+	695 (55.8%)	4730 (41.2%)	5425 (42.6%)

Number of patients (percentage) within each group. Polytrauma is defined by the “Berlin definition”. (18)

AIS = abbreviated injury scale; BP = blood pressure; DDI = delayed diagnosed injuries, GCS = Glasgow Coma Scale, ICU = intensive care unit; ISS = injury severity score; N = number of patients; PRBC = packed red blood cells; SD = standard deviation.

While DDI generally comprised all body regions, the top 10 of DDIs are shown in Table 2. The majority of injuries diagnosed after the initial trauma room admission affected the head, and even comprised severe head injuries like subarachnoid hemorrhage. While most missed injuries to the extremities were distal (e.g. carpal/metacarpal), truncal injuries (e.g. thoracic spine) were also missed during the initial evaluation.

**Table 2:** Top 10 of initially missed injuries. Numbers and percentages refer to 60,100 diagnoses, not to patients. These 10 diagnoses account for 24% (428/1809) of all delayed diagnosed injuries. LOC: loss of consciousness.

Rank	Description / Body region	Number	Percent
	Brain Injury		
1	Diffuse axonal injury with LOC 6–24 h	74	4.1%
2	Diffuse axonal injury with LOC > 24 h	66	3.6%
5	Brain swelling/edema: moderate; compressed ventricles and brain stem cisterns	37	2.0%
7	Subarachnoid hemorrhage	31	1.7%
7	Brain swelling/edema: mild; compressed ventricles without compressed brain stem cisterns	31	1.7%
10	Brain swelling/edema: severe; absent ventricles or brain stem cisterns	30	1.7%
	Upper extremities		
4	Bony injury/fracture of metacarpal bone	38	2.1%
7	Bony injury/fracture of carpal bone	31	1.7%
	Lower extremities		
3	Tendon/ligament injury	54	3.0%
	Spine		
6	Thoracic spine fracture with or without dislocation, but no cord involvement	36	2.0%

**Table 3:** Logistic regression analysis

	Odds Ratio (OR)	95% confidence interval for OR	*p*-value
Number of diagnoses	1.19	1.16–1.22	< 0.001
Transferred-in	0.63	0.50–0.80	< 0.001
Hospital level 2/3 ^1^	1.89	1.61–2.22	< 0.001
Worst injury severity^2^			< 0.001
AIS 3	1.24	1.03–1.50	
AIS 4	1.73	1.40–2.13	
AIS 5	2.15	1.70–2.72	
AIS 6	2.71	1.39–5.29	
Injured body regions (AIS 2+)			
Head	1.14	0.98–1.32	0.089
Thorax	0.98	0.84–1.13	0.74
Abdomen	1.23	1.06–1.42	0.006
Spine	0.86	0.74–1.00	0.046
Arms	1.03	0.90–1.19	0.65
Legs	1.25	1.08–1.43	0.002
Pelvis	0.75	0.64–0.89	< 0.001
Computed tomography	0.64	0.53–0.78	< 0.001
GCS ≤ 8	1.09	0.92–1.3	0.33
Age^3^			0.14
0–2	0.91	0.67–1.24	0.55
3–5	1.18	0.90–1.56	0.23
6–10	0.96	0.77–1.19	0.70
11–15	0.83	0.69–0.99	0.04

AIS = Abbreviated Injury Scale; GCS = Glasgow Coma Scale.

*Reference values or group*: ^*1*^ *reference: Level 1*, ^*2*^ *reference: AIS 2*,^*3*^ *reference: 16–20 years*.

### Management of delayed diagnosed injuries

We identified a total of 1,809 DDIs in this study, 33.5% (606 injuries) of which necessitated surgical intervention. In comparison, primarily correctly identified injuries required surgery in 36.1%.

The individual injury severities of a DDI were also analyzed, and we found that surgery was required for a DDI (non-DDI) with an AIS 1 = 22% (23%), AIS 2 = 28% (32%), AIS 3 = 42% (45%), AIS 4 = 42% (47%) and AIS 5 = 44% (65%), respectively.

We further analyzed the occurrence of DDIs under specific circumstances.

### Age

The DDI-group had a mean age of 15.1 ± 5.3 years, while the no-DDI group had a mean age of 14.6 ± 5.5 years. After allocation of patients to different age groups the rate of DDI within each group was: 0–2 years old (53 patients; 8%); 3–5 years (68 patients; 9.8%); 6–10 years (112 patients; 8.4%); 11–15 years (168 patients; 7.9%); 16–20 years (845 patients; 10.7%).

**Fig. 1:**
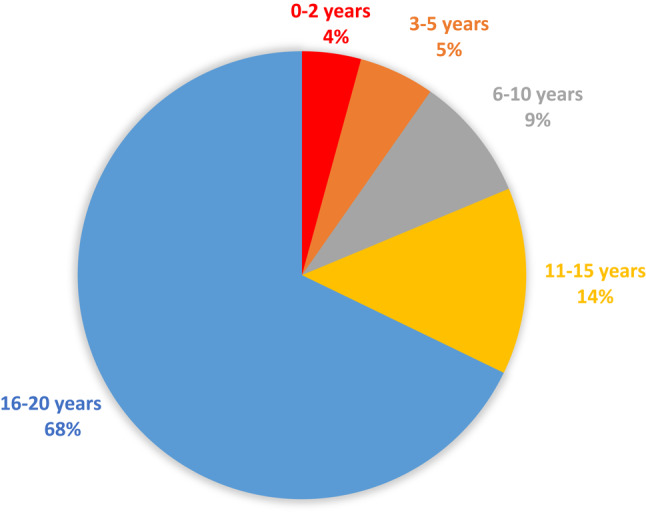
Distribution of patients with delayed diagnosed injuries per defined age group. Overall population with DDI: *n* = 1246.

The older patients were the more they contributed to the group of delayed diagnosed injured patients. The distribution of patients with DDI by age is depicted in Fig. 1.

### Mechanism of injury

Overall, car and motorcycle accidents were the most common cause for hospital admission in our study, as they were the predominant injury mechanisms in the DDI group. **(**Table 1**)**

**Fig. 2:**
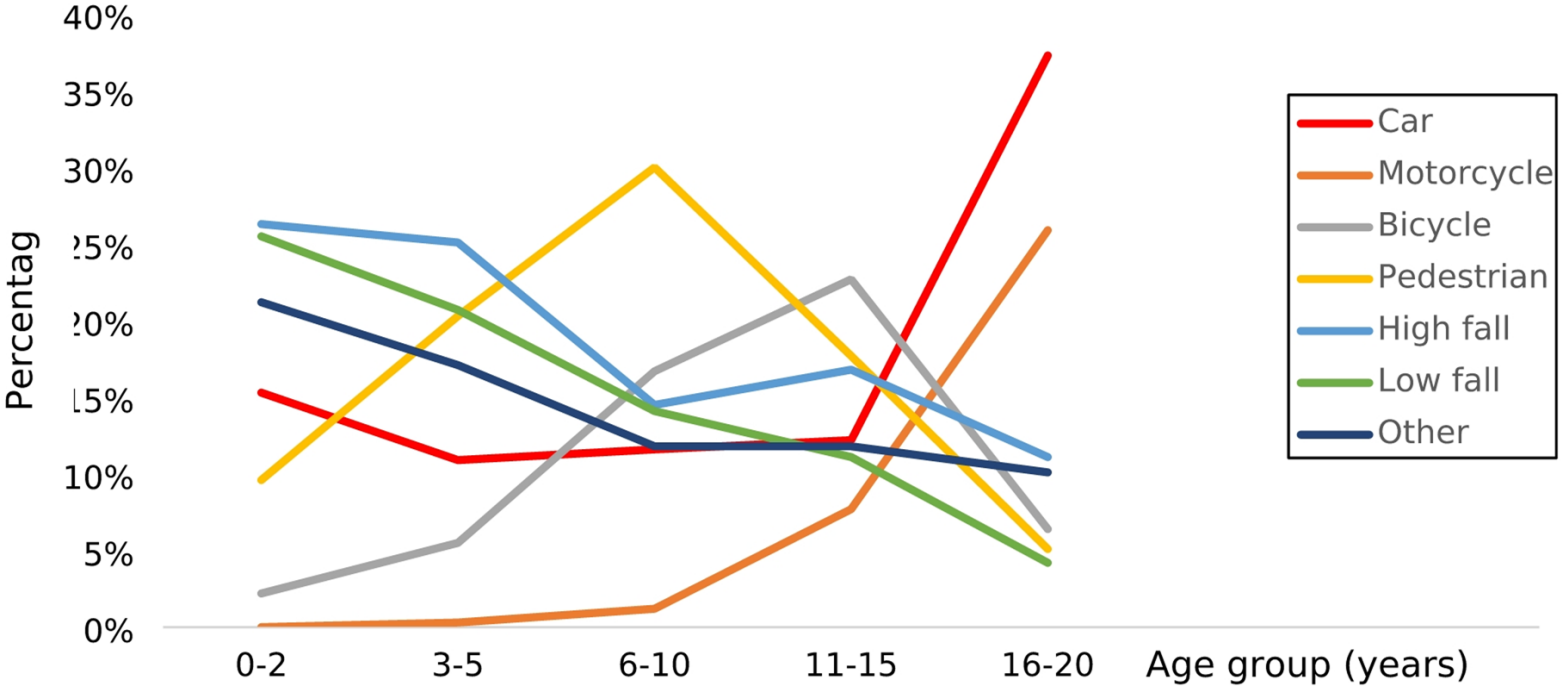
Age dependent change of injury mechanism and percentage of each mechanism within the respective age group. Overall population *n* = 12,562. No injury mechanism was recorded in 171 patient files.

The predominant mechanism of injury changed depending on patient age. While falls were the most common causes of accidents in children up to 5 years, traffic-related injuries, particularly those caused by cars, bicycles and motorcycles, became most important for children aged 11 years and older (Fig. 2).

### Injury severity

Patients requiring intubation before hospital admission showed a significantly higher incidence of DDI than those not needing intubation (13.6% vs. 8.2%; *p* < 0.001). Unconscious patients with a GCS ≤ 8 were more often associated with DDIs (14.9%) compared to the DDI rate in patients with a GCS > 8 (8.7%). Injuries to the thorax and head were most frequently diagnosed delayed, followed by limb injuries. DDIs to the head had a significantly higher mean abbreviated injury score (AIS_Head_) than the no-DDI group (3.6 vs. 2.7; *p* < 0.001).

The ISS in the DDI group was significantly higher than the no-DDI group’s (24.1 ± 14.5 vs. 17.5 ± 12.2; *p* < 0.001). Additionally, the proportion of initially missed injuries also rose with the injury severity. Furthermore, we found that polytraumatized patients, defined by the “Berlin definition”^18^ carried a significantly higher risk of initially missed injuries (17.1%) compared to non-polytraumatized patients (8.4%) (*p* < 0.001) (Table 1).

Additionally, the proportion of DDIs increased in alignment with the number of diagnoses. Data show that the number of diagnoses made has a direct impact on the rate of DDIs. Specifically, in the patient group with one diagnosed injury, 4.8% of those injuries were initially missed. This number rose to even 19.1% of initially missed injuries in the patient subgroup in whom seven injuries had been diagnosed. (*p* < 0.001)

**Fig. 3:**
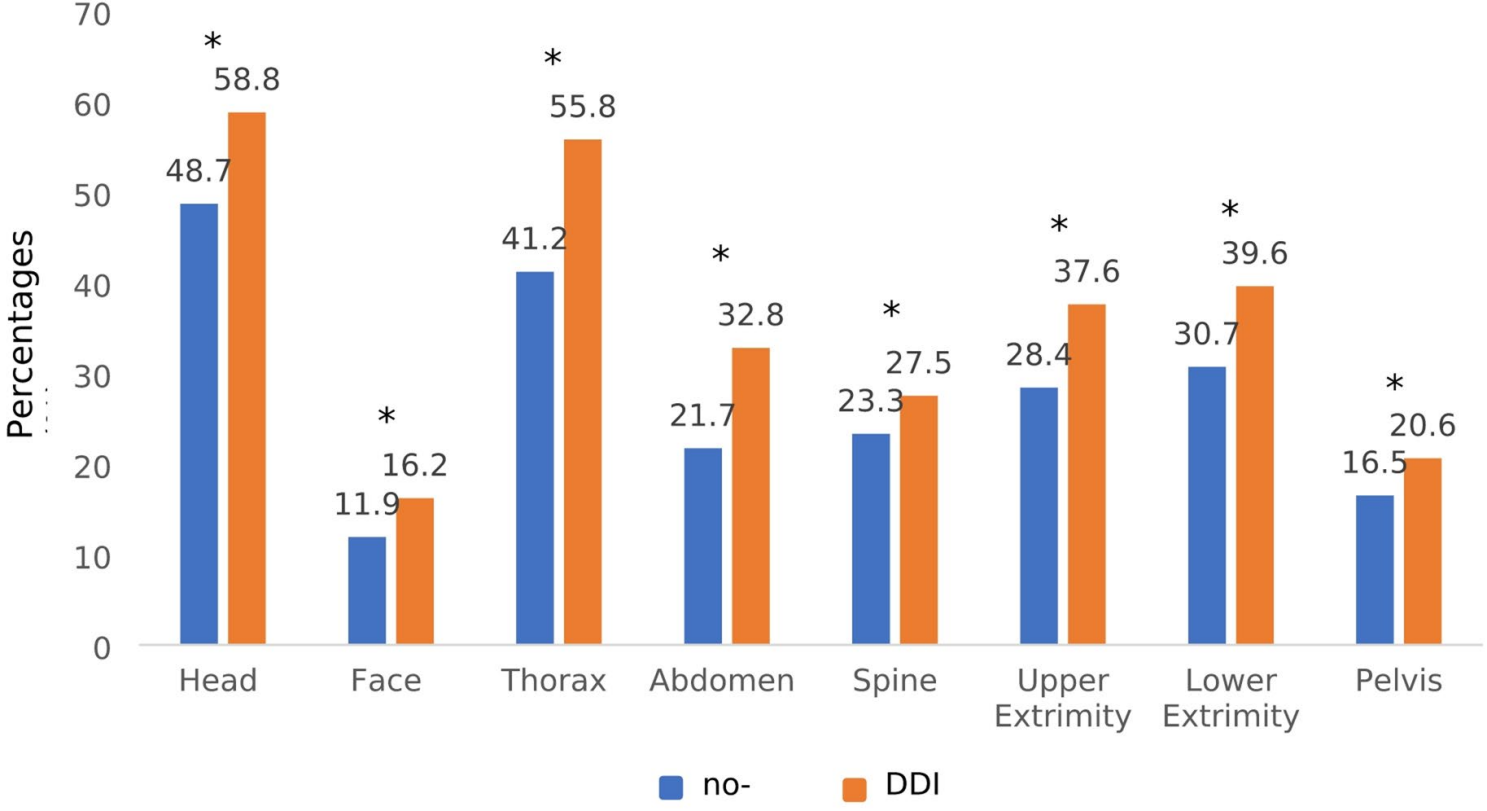
Rate of relevant injuries (AIS ≥ 2) to the respective body region in the DDI and no-DDI group. Population *n* = 1246 (DDI) and *n* = 11,487 (no-DDI). *p < 0.001 within every body region.

Comparing the two groups considering every body region depicted, the DDI group revealed a significantly higher percentage of relevant injuries (AIS ≥ 2) within the delayed diagnosed injuries compared to the rate of relevant injuries found in the respective no-DDI group. (Fig. 3)

### Imaging

Sonographic examination was frequently conducted (> 90%) within both patient groups (DDI and no-DDI group), regardless of the injury severity. Also, we observed no significant difference in the rate of acquired plain radiological imaging between the two groups during the initial trauma room management. Although the utilization of whole-body computed tomography increased with injury severity, the difference in the rate of initially missed injuries with or without WBCT remained < 2% in all ISS subgroups. The DDI group showed an even higher rate of delayed diagnosed injuries in patients who initially underwent a WBCT scan (10.2%) than patients without WBCT (8.7%). The rate of initially missed injuries in the subgroup of polytraumatized children receiving an initial WBCT was > 17%.

### Hospital stay

The DDI group revealed a longer stay in the intensive care unit (9.4 ± 11.7 days) and in the hospital (18.8 ± 17.5 days) than the no-DDI group (5.6 ± 9.1 and 13.9 ± 15.5 days, respectively).

### Organizational aspects

Percentages of patients in whom diagnoses were initially missed did not differ significantly according to whether they were treated in the trauma room during the day, at night, on weekdays or on weekends. There was also no significant difference in percentages of patient**s** with DDIs who were cared for at a single designated hospital and those who were transferred to another (9.9% vs. 8.6%). However, hospitals providing a lower level of care and not transferring injured children demonstrated a significantly higher risk of initially missing injuries than did level 1 trauma centers (OR 1.89; CI 1.61–2.22; *p* < 0.001). The percentage of patients with DDIs was 14.5%, 12.8%, and 9.2% in the Level 3, 2, and 1 trauma centers, respectively.

### Risk factors for missed injuries

Logistic regression analysis showed that the number of injuries raised the risk of initially missing injuries. Each injury increased the risk of having a DDIs by about 20% (OR 1.19; CI 1.16–1.22; *p* < 0.001). Additionally, the more severe the worst injury was, the higher the likelihood of an initially missed injury. This was especially true in patients suffering from relevant injuries to the abdomen (OR 1.23; CI 1.06–1.42; *p* = 0.006) or lower extremities (OR 1.25; CI 1.08–1.43; *p* = 0.002).

Treatment of patients in hospitals not assigned as Level 1 hospitals significantly increased the DDI risk (OR 1.89; CI 1.61–2.22; *p* < 0.001). Once patients were transferred to another hospital, the DDI risk was lower than in primarily admitted patients.

When patients were diagnosed via a computed tomography scan, even a limited scan (e.g. cranial CT) resulted in significantly lowering the risk of DDIs.

On the other hand, neither patient age nor unconsciousness (GCS ≤ 8) proved to be independently associated with an increased risk for a DDI during the initial treatment.

Overall results of our logistic regression analysis are shown in Table 3.

## Discussion

Despite a thoughtful, structured approach, including a thorough clinical examination, several studies have confirmed that significant injuries are being missed during initial management^19–21^. The issue of DDIs is especially important in the context of treating severely injured children, but reliable data in this specific patient population is scarce. Hence, we sought to characterize delayed diagnosed injuries and accompanying risk factors in the severely injured children and adolescent patient population.

Rates of initially DDIs not specific to children and adolescent populations, vary in the literature. As far as clinically significant DDIs are concerned, reported rates range from 15% − 22%^19,20,22^. Furthermore, 27% − 66% of unrecognized diagnoses were major injuries^19,23,24^. This wide range is partially attributable to different patient populations and how DDIs are defined^19^.

It is inherent to the nature of acute trauma care that young children cannot adequately communicate their pain and injuries due to their developmental stage. However, our findings reveal that age is not an independent risk factor for a DDI. Compared to the existing literature in all-age patient populations, our DDI rate is well within the range of these recently reported ones from 1.5% − 18.3%^25–27^. Interestingly, a lower GCS of ≤ 8 in obtunded to comatose children in this study was not a risk factor for initially missing an injury in our logistic regression analysis either.

Independent risk factors for DDIs during the initial hospital treatment period and thus delaying the diagnosis of an injury beyond the trauma room in our patient population were: a higher number of injuries, overall injury severity (ISS), and an AIS > 2 in the abdominal and lower extremity body region. In the literature, several of these have already been recognized as risk factors in the general trauma patient population^19,27–30^. Diagnosed injuries to the pelvis and spine were associated with a lower risk of DDI in our population. One possible explanation is that these patients underwent computed tomography scans and were therefore thoroughly examined. The widespread availability of Multiple Slice Computed Tomography (MSCT) scans and integration of computed tomography (CT) in the emergency room has greatly improved diagnostic procedures and has resulted in injuries being detected earlier^19,31^. Since the diagnostics of critically injured patients must focus on life-threatening injuries, the pelvis is usually scanned during combined abdomen/pelvis CT examination^32^. This is in line with our patient population, where we report the benefit of applied computed tomography scan in significantly lowering the risk of initially missing injuries. However, our reported rate of WBCT is higher than published protocols in German polytraumatized children^33^. Yet, in our study, we included adolescents up to 20 years of age where WBCT is used similar to adult treatment protocols during initial patient work-up, resulting in higher rates of WBCT.

Our apparently contradicting results that CTs are associated with a lower DDI risk in multivariate analysis, but higher DDI rate in WBCT group can be explained, because this is mainly due to more severe cases in the WBCT group. Multivariate analysis on the other hand adjusts for severity; thus, the (adjusted) effect of performing a CT is that the risk of DDI is lower when this diagnostic is applied.

There is evidence that a delayed diagnosis or misdiagnoses in multiple trauma patients delays effective treatment, prolongs hospital stay, and increases mortality^22^. Similarly, our findings indicate that a significant proportion of DDIs in our patient population involve the head, with an AIS_Head_ severity rating ranging from 1 to 6. Reportedly, head injuries can be as high as 35.8% among DDIs^27^. It is evident that children who have sustained severe injuries have also frequently sustained head injuries. Noteworthy, in our study, DDIs to the head were more severe. This might indicate that the severity of the head injury had only developed partially and was not entirely apparent on the primary admission. However, because of the retrospective data we evaluated, it is unclear whether the “newly” documented injury in the intensive care unit is, in fact, an initially missed injury or rather a secondary injury developing or worsening from the primary injury to the head region. Just as stated before - that relatively high numbers of patients with a head DDI raises the question of whether routine follow-up CT scans could close a diagnostic gap, since subdural hematomas can develop within 24 h to 48 h after trauma^27^. This also includes an axonal shear injury detected during an MRI extension of diagnostics, which is not assessable via initial trauma room imaging; it would then be considered as a newly found and therefore delayed diagnosed injury. Furthermore, trauma room diagnostics are frequently reassessed in the intensive care unit, a factor that can also lead to a new diagnosis based on a secondary radiological reading of images. Banaste et al. demonstrated that a re-evaluation of initial radiological findings as part of a WBCT of 2354 severely injured patients by a second radiologist identified 12.9% of initially missed injuries^34^. This might also help to explain why such a high proportion of DDIs is possible despite initial CT scans.

Our study reveals a higher risk of initially missed injuries in children and adolescents who were not treated in a level 1 center. The majority of studies involving severely injured children demonstrate superior mortality and morbidity outcomes for those treated in a designated pediatric trauma center (PTC) than in an adult trauma center (ATC), particularly younger children^35–38^. A recent meta-analysis by Moore et al. demonstrated that, in addition to a reduction in mortality, the subgroup of children with head injuries and penetrating injuries who were treated in a pediatric trauma center revealed a superior outcome^39^. A recent Dutch study showed that severe pediatric trauma in the Netherlands was predominantly treated in Level I trauma centers, where a multidisciplinary team of experts is available. The authors state that raising the numbers of severely injured patients brought primarily to level-1 trauma centers may help to reduce mortality further^40^. In Germany, according to the guideline for certification of trauma centers of the DGU, the involvement of specialized pediatric trauma surgeons and pediatric critical care units in the care of pediatric trauma patients is mandatory in Level I centers, just as is the rapid availability of a multidisciplinary team in the trauma room. These factors may explain why our results suggest that a better interdisciplinary infrastructure and higher standards in trauma room care lower the risk of initially missing injuries.

However, these hypotheses cannot be answered with the present data and should be addressed in further investigations or longitudinal studies.

Delayed diagnosed injuries have different impacts on a patient, depending on the necessary treatment. If the treatment remains non-operative, the impact on the clinical course should be minimal, besides potentially prolonging the hospital stay. In case of surgical treatment, multiple parameters (e.g. length of ICU or hospital stay, complication rate, etc.) could be affected. Our reported rate of surgical interventions in DDIs is comparable to the published literature (31.4%) and emphasizes the importance of this issue^21^. The influences of DDI on the course of intensive care treatment, length of stay on the ICU and/or in hospital differ presumably for DDI affected body regions. Predominantly DDI to the head, abdomen, thorax and spine have the potential to prolong and complicate ICU treatment and hospital stay.

A structured clinical examination of the seriously injured patient is mandatory during the initial assessment. This was stated by the American College of Surgeons Committee on Trauma using the ATLS^®^ concept. The initial approach consists of two diagnostic pillars in the trauma room: the “primary survey” and the “secondary survey”^41^.

In cases of a severe injury in children, injuries to the head or trunk are often of primary concern and injuries to the distal limbs are therefore typically given less attention during the primary and possibly even secondary survey. It is then essential to conduct the tertiary survey in the ICU or regular ward as soon as possible. This should be done following a standard protocol and as thoroughly as possible. Despite the widespread adoption of the structured tertiary survey at most trauma centers, there is considerable variation in the documentation of this process. As a result, the present data can provide no information about the timepoint at which a DDI was detected, or the latency between the initial examination and subsequent discovery of the injury. Nevertheless, there is ample evidence that an institutionalized tertiary trauma survey is important to detect injuries in a timely manner to be able to treat the DDI without further harm to the patient^25,42^. In the long term, injuries that have been missed can often overshadow successful life-saving measures and may have legal consequences. Therefore, once serious injuries have been ruled out, the secondary survey should be conducted at the conclusion of the trauma room phase to detect as many injuries as possible. This procedure must also be followed in patients who have been transferred. Regular evaluation throughout the treatment course and during the tertiary survey are of particular importance in the intensive care unit and later on the regular ward, as missed injuries and gradually evolving secondary injuries may be identified with a time lag and from a different perspective.

In contrast to previous studies of delayed diagnosed injuries in children, we focused on identifying risk factors potentially leading to missing injuries during the initial treatment. A higher ISS had been reported before in patients suffering from orthopedic injuries and sustaining DDIs, corresponding to longer hospital stays^43,44^. Yet, our findings indicate that not only the total ISS is a risk factor, so is the worst injury (AIS > 2) in any region itself an independent and with the severity (AIS) increasing risk for missing injuries. Interestingly, Choi et al.^44^ found the ISS to be an independent risk in injured children for unplanned re-admission - a finding which is beyond the scope of our data.

### Limitations

Several factors in this study must be interpreted carefully. Registry data are generally less valid than data provided by prospective randomized controlled studies. The actual time point of diagnosis is not documented in the TR-DGU and we can assume that not all DDIs were documented in the TR-DGU as this has to be actively marked in the web-based interface. Furthermore, diagnoses are documented as AIS codes, which limit the accuracy of DDI since no unequivocal code exists for each type of injury.

The secondary survey is usually carried out at the end of the trauma room care. However, it is not uniformly defined in the literature. The tertiary survey, which is mostly carried out in the ICU is often referred to as a secondary survey. Therefore, the timeslot is unclear from the available data as to when precisely the injuries were identified as initially missed injuries.

Despite the comprehensive nature of the collected data, it does not provide the necessary information for all inquiries. It is impossible to determine whether the outcome was affected by the undetected injuries or the delayed diagnosis. Monocentric data collection is still necessary, as the data that is available only enables limited tracing of the treatment delivered.

More studies are needed to investigate the burden of delayed diagnosed injuries in pediatric and adolescent trauma patients, with the aim of alleviating the consequences.

## Conclusion

The present study demonstrates that injuries in pediatric and adolescent trauma patients are frequently missed in the context of trauma room diagnostics. We have identified risk factors for missed injuries such as growing numbers of trauma diagnoses, higher injury severity scores, and treatment in level 2 or 3 trauma centers. According to our findings, transferred patients and patients receiving CT scans have a lower risk for an injury being missed. Most of the time, head injuries are often diagnosed late, which is potentially a reason to perform cranial CT or MRI even after initial trauma room assessment on clinical judgement. Other often missed injuries belong to distal and lower extremity regions. Searching for initially missed injuries in a standardized tertiary trauma survey is justified, as a significant number of missed injuries still require surgical treatment.

## Data Availability

The datasets used and/or analyzed during the current study are available from the corresponding author on reasonable request and with permission of the TraumaRegister DGU (TR-DGU) of the German Trauma Society (DGU).

## References

[1] Eimer, C., Buschmann, C., Deeken, J. & Kerner, T. Mechanical trauma in children and adolescents in Berlin. Forensic Sci Med Pathol. Apr 16. (2024). 10.1007/s12024-024-00814-7. Epub ahead of print. PMID: 38625460. 10.1007/s12024-024-00814-7 PMC11953125 38625460

[2] Schuster, A. et al. Injury pattern and current early clinical care of pediatric polytrauma comparing different age groups in a level I trauma center. *J. Clin. Med.* **13** (2), 639. 10.3390/jcm13020639 (2024). PMID: 38276145; PMCID: PMC10816860. 38276145 10.3390/jcm13020639 PMC10816860

[3] Cockrell, H. C. & Greenberg, S. L. M. General care considerations for the pediatric trauma patient. *Oral Maxillofac. Surg. Clin. North. Am.* **35** (4), 493–499 (2023). 37625944 10.1016/j.coms.2023.05.003

[4] German Federal Statistical Office. Child road traffic accidents in 2018, 2019. Apr 1;1–59.

[5] Meier, R. et al. The multiply injured child. *Clin. Orthop. Relat. Res.* **432** (NA;), 127–131 (2005). 10.1097/01.blo.0000156005.01503.0a 15738812

[6] Waydhas, C. Advanced trauma life support. *Notf Rettungsmedizin*. **6** (1), 33–36 (2003).

[7] Snyder, C. L. et al. Blunt trauma in adults and children: a comparative analysis. *J. Trauma: Injury Infect. Crit. Care*. **30** (10), 1239–1245 (1990 Oct). 10.1097/00005373-199010000-00008 2213932

[8] Scaife, E. R. & Rollins, M. D. Managing radiation risk in the evaluation of the pediatric trauma patient. *Semin Pediatr. Surg.* **19** (4), 252–256 (2010). 20889080 10.1053/j.sempedsurg.2010.06.004

[9] Lehner, M., Jung, P., Olivieri, M & Schmittenbecher, P. P. Multiple trauma care in childhood — practical and pragmatic summary of the new guideline. *Notfall Rettungsmed*. **24**, 32–42. 10.1007/s10049-020-00830-4 (2021).

[10] Muhm, M., Danko, T., Henzler, T., Luiz, T., Winkler, & H. Ruffing, T. Pediatric trauma care with computed tomography—criteria for CT scanning. *Emerg. Radiol.* **22** (6), 613–621 (2015). 26208818 10.1007/s10140-015-1332-7

[11] Hilbert-Carius, P. et al. Whole-body-CT in severely injured Children. Results of Retrospective, multicenter study with patients from the traumaregsiter DGU^®^. *Klin. Padiatrie*. **227** (4), 206–212 (2015). 10.1055/s-0035-1547311 25875400

[12] Berger, M. et al. Mortality with and without whole-body CT in severely injured children. *Dtsch. Ärzteblatt Int.* **120** (11), 180–185 (2023). 10.3238/arztebl.m2022.0414 PMC10213478 36633453

[13] Abe, T. et al. Is Whole-Body CT associated with reduced In-Hospital mortality in children with trauma? A nationwide study. *Pediatr. Crit. Care Med.* 2019;Publish Ahead of Print(NA;):NA;. 10.1097/PCC.0000000000001898 30730378

[14] Giannakopoulos, G. F. et al. Missed injuries during the initial assessment in a cohort of 1124 level-1 trauma patients. *Injury* **43** (9), 1517–1521 (2012). 21820114 10.1016/j.injury.2011.07.012

[15] Suda, A. J. et al. Delayed diagnosed trauma in severely injured patients despite guidelines-oriented emergency room treatment: there is still a risk. *Eur. J. Trauma. Emerg. Surg.* **48** (3), 2183–2188 (2022). 34327544 10.1007/s00068-021-01754-5 PMC9192381

[16] Kim, S. et al. Detection of missed fractures of hand and forearm in whole-body CT in a blinded reassessment. *BMC Musculoskelet. Disord*. **22** (1), 589 (2021). 34174869 10.1186/s12891-021-04425-z PMC8236191

[17] Bahramian, M., Shahbazi, P., Hemmati, N., Mohebzadeh, P. & Najafi, A. Extremity fractures as the most common missed injuries: A prospective cohort in intensive care unit admitted multiple trauma patients. *Indian J. Crit. Care Med: Peer-Rev Off Publ Indian Soc. Crit. Care Med.* **27** (3), 201–204 (2023). 10.5005/jp-journals-10071-24426 PMC10028718 36960108

[18] Pape, H. C. et al. The definition of polytrauma revisited: An international consensus process and proposal of the new ‘Berlin definition’. J Trauma Acute Care Surg. ;77(5):780–786. (2014). 10.1097/TA.0000000000000453. PMID: 25494433. 10.1097/TA.0000000000000453 25494433

[19] Pfeifer, R. & Pape, H. C. Missed injuries in trauma patients: A literature review. *Patient Saf. Surg.* **2** (1), 20–20 (2008). 18721480 10.1186/1754-9493-2-20 PMC2553050

[20] Buduhan, G. & McRitchie, D. I. Missed injuries in patients with multiple trauma. J Trauma. ;49(4):600-5. (2000). 10.1097/00005373-200010000-00005. PMID: 11038075. 10.1097/00005373-200010000-00005 11038075

[21] Houshian, S., Larsen, M. S. & Holm, C. Missed injuries in a level I trauma center. *J. Trauma: Inj Infect. Crit. Care*. **52** (4), 715–719 (2002). 10.1097/00005373-200204000-00018 11956389

[22] Janjua, K. J., Sugrue, M. & Deane, S. A. Prospective evaluation of early missed injuries and the role of tertiary trauma survey. *J. Trauma: Inj Infect. Crit. Care*. **44** (6), 1000–1007 (1998). 10.1097/00005373-199806000-00012 9637155

[23] Juhl, M., Møller-Madsen, B. & Jensen, J. Missed injuries in an orthopaedic department. Injury. ;21(2):110-2. (1990). 10.1016/0020-1383(90)90067-5. PMID: 2351463. 10.1016/0020-1383(90)90067-5 2351463

[24] Kremli, M. K. Missed musculoskeletal injuries in a University Hospital in Riyadh: types of missed injuries and responsible factors. Injury. ;27(7):503-6. (1996). 10.1016/0020-1383(96)00044-7. PMID: 8977838. 10.1016/0020-1383(96)00044-7 8977838

[25] Wilbers, A. et al. An analysis of missed injuries at a level 1 trauma center with a tertiary survey protocol. *Am. J. Surg.* **224** (1), 131–135 (2022). 35440377 10.1016/j.amjsurg.2022.04.010

[26] Selçuk, H., Oray, N., Mert, R. M., Odaman, H. & Güleryüz, H. Evaluation of missed radiological diagnosis in multiple trauma patients with Full-Body computed tomography in the emergency department. *Cureus* **16** (1), e51621 (2024). 38318559 10.7759/cureus.51621 PMC10839344

[27] Gümbel, D., Matthes, G., Ekkernkamp, A., Laue, F. & Lefering, R. Influencing factors for delayed diagnosed injuries in multiple trauma patients – introducing the ‘Risk for delayed diagnoses score’ (RIDD-Score). *Eur. J. Trauma. Emerg. Surg.* ;1–9. (2024). 10.1007/s00068-024-02571-2 PMC11599345 38926171

[28] Ferree, S. et al. Tertiary survey in polytrauma patients should be an ongoing process. *Injury* **47** (4), 792–796 (2016). 26699429 10.1016/j.injury.2015.11.040

[29] Thomson, C. B. & Greaves, I. Missed injury and the tertiary trauma survey. *Injury* **39** (1), 107–114 (2008). 18164007 10.1016/j.injury.2007.07.030

[30] Chen, C. W., Chu, C. M., Yu, W. Y., Lou, Y. T. & Lin, M. R. Incidence rate and risk factors of missed injuries in major trauma patients. *Accid. Anal. Prev.* **43** (3), 823–828 (2011). 21376872 10.1016/j.aap.2010.11.001

[31] Hessmann, M. H., Hofmann, A., Kreitner, K., Lott, C. & Rommens, P. M. The benefit of multislice computed tomography in the emergency room management of polytraumatized patients. *Eur. J. Trauma.* **31** (3), 231–238 (2005). 10.1080/00015458.2006.11679940 17168258

[32] Falchi, M. & Rollandi, G. A. CT of pelvic fractures. *Eur. J. Radiol.* **50** (1), 96–105 (2004). 15093240 10.1016/j.ejrad.2003.11.019

[33] Bayer, J., Reising, K., Kuminack, K., Südkamp, N. P. & Strohm, P. C. Is Whole-Body computed tomography the standard Work-up for Severely-Injured children? Results of a survey among German trauma centers. *Acta Chir. Orthop. Traumatol. Cech*. **82** (5), 332–336. 10.55095/achot2015/055 (2015). 26516949

[34] Banaste, N. et al. Whole-Body CT in patients with multiple traumas: Factors leading to missed injury. *Radiology* **289** (2), 374–383 (2018). 30084754 10.1148/radiol.2018180492

[35] McCarthy, A., Curtis, K. & Holland, A. J. A. Paediatric trauma systems and their impact on the health outcomes of severely injured children: An integrative review. *Injury* **47** (3), 574–585 (2016). 26794709 10.1016/j.injury.2015.12.028

[36] Sathya, C. et al. Mortality among injured children treated at different trauma center types. *JAMA Surg.* **150** (9), 874–881 (2015). 26106848 10.1001/jamasurg.2015.1121

[37] Potoka, D. A. et al. Impact of pediatric trauma centers on mortality in a statewide system. *J. Trauma: Inj Infect. Crit. Care*. **49** (2), 237–245 (2000). 10.1097/00005373-200008000-00009 10963534

[38] Notrica, D. M. et al. Pediatric trauma centers. *J. Trauma. Acute Care Surg.* **73** (3), 566–572 (2012). 22929485 10.1097/TA.0b013e318265ca6f

[39] Moore, L. et al. Pediatric vs adult or mixed trauma centers in children admitted to hospitals following trauma. *JAMA Netw. Open.* **6** (9), e2334266 (2023). 37721752 10.1001/jamanetworkopen.2023.34266 PMC10507486

[40] Fylli, C., Schipper, I. B. & Krijnen, P. Pediatric trauma in the netherlands: Incidence, mechanism of injury and In-Hospital mortality. *World J. Surg.* **47** (5), 1116–1128 (2023). 36806556 10.1007/s00268-022-06852-y PMC10070213

[41] American College of Surgeons’ Committee on Trauma; International ATLS working group. Advanced trauma life support (ATLS^®^): the ninth edition. *J. Trauma. Acute Care Surg.* **74** (5), 1363–1366 (2013). 23609291 10.1097/TA.0b013e31828b82f5

[42] Parson, M., Pickard, A., Simpson, D., Treece, M. & Rampersad, L. UK-wide major trauma center tertiary trauma survey pro forma review and aggregation and consolidation into a redesigned document. *Trauma. Surg. Acute Care Open.* **8** (1), e000903 (2023). 36632529 10.1136/tsaco-2022-000903 PMC9827263

[43] Podolnick, J. D., Donovan, D. S. & Atanda, A. W. Jr. Incidence of delayed diagnosis of orthopaedic injury in pediatric trauma patients. *J. Orthop. Trauma.* **31** (9), e281–e287. 10.1097/BOT.0000000000000878 (2017). 28471915 10.1097/BOT.0000000000000878

[44] Choi, P. M., Yu, J. & Keller, M. S. Missed injuries and unplanned readmissions in pediatric trauma patients. *J. Pediatr. Surg.* **52** (3), 382–385. 10.1016/j.jpedsurg.2016.10.005 (2017). 27839721 10.1016/j.jpedsurg.2016.10.005 PMC5409520

